# Atomic structure and passivated nature of the Se-treated GaAs(111)B surface

**DOI:** 10.1038/s41598-018-19560-2

**Published:** 2018-01-19

**Authors:** Akihiro Ohtake, Shunji Goto, Jun Nakamura

**Affiliations:** 1 National Institute for Materials Science (NIMS), Tsukuba, 305-0044 Japan; 2 Department of Engineering Science, The University of Electro-Communications (UEC-Tokyo), Chofu, Tokyo, 182-8585 Japan

## Abstract

We have systematically studied the atomic structure and electronic properties of the Se-treated GaAs(111)B surface using scanning tunneling microscopy, reflection high-energy electron diffraction, x-ray photoelectron spectroscopy, and first-principles calculations. We have found that Se atoms substitute $\frac{3}{4}$ monolayer of As atoms at the outermost layer of the ideal (111)B surface. Charge transfer from Se to As eliminates all of unsaturated dangling bonds, so that the surface is electronically stabilized, leaving no surface states in the mid-gap region.

## Introduction

The electronic properties of GaAs surfaces are characterized by the existence of high density of surface states that pin the Fermi level at midgap. For the improvement of electronic properties of GaAs surfaces and the development of GaAs-based devices, it is necessary to reduce the surface-state density and to control the Fermi-level position. Passivation of GaAs surfaces with either S or Se atom has been shown to be very effective in reducing surface states, which leads to the dramatic improvement in surface-dependent properties of GaAs^1–6^.

There has recently been revived interest in the use of the S and Se passivation for the improvement of GaAs-based devices^7–10^. On the other hand, despite considerable efforts in the last three decades, the mechanism of the passivation induced by S/Se treatments is still far from being completely understood. To understand the passivation mechanism, information about the structural and electronic properties of the S/Se-treated GaAs surface is required.

**Figure 1:**
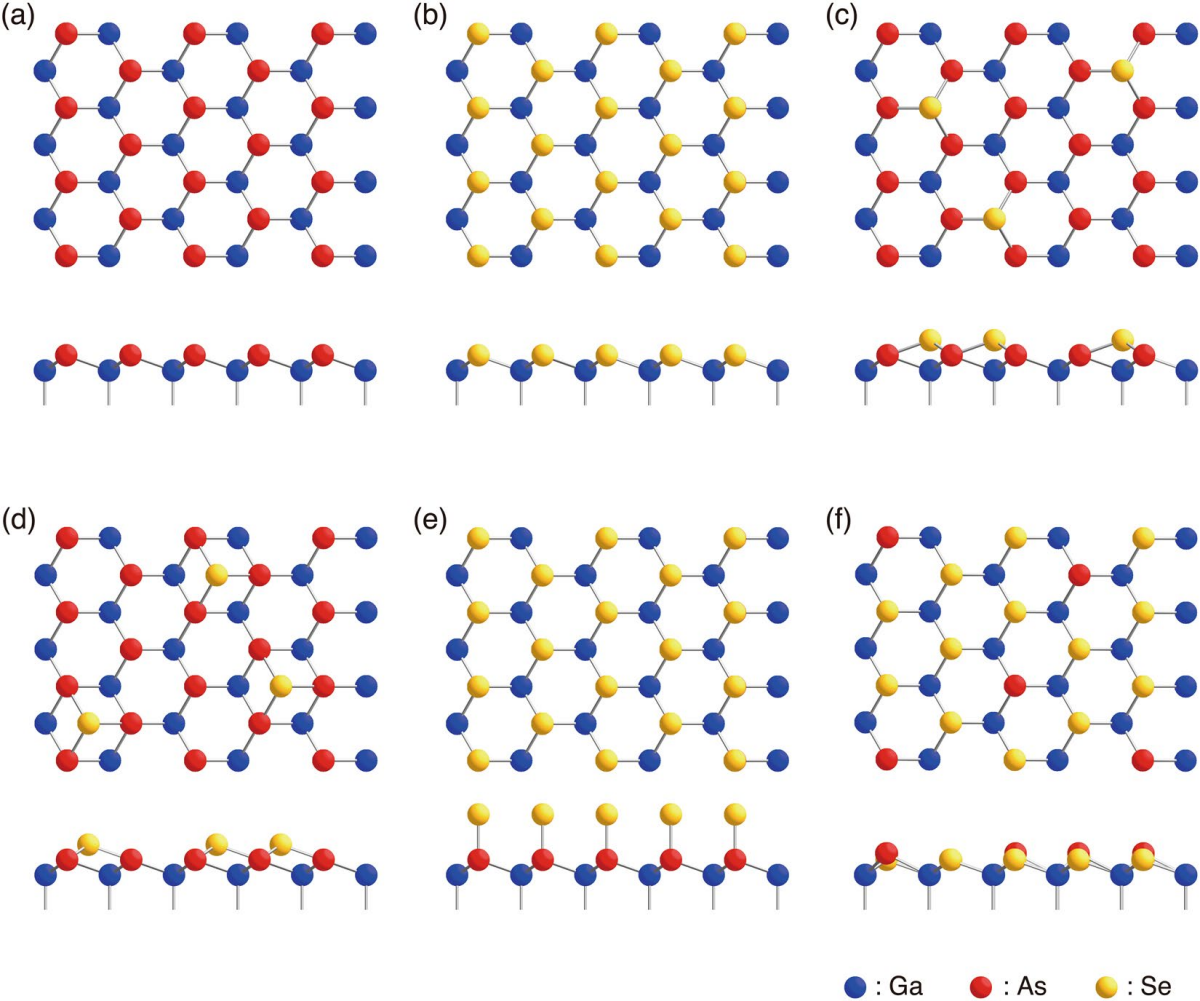
Top and side view of structure models for the Se-treated GaAs(111)B-(1 × 1) surface; (**a**) ideal As-terminated model, (**b**) Se-terminated model, (**c**) T_4_-site model, (**d**) H_3_-site model, (**e**) on-top site model, and (**f**) mixed Se/As-terminated model.

This paper reports a combined experimental and theoretical study on the structure and electronic properties of Se-treated GaAs(111)B surface. Previous studies have shown that the GaAs(111)B surfaces treated by S and Se show a (1 × 1) symmetry^11–13^. A structure model has been proposed, in which the outermost As atoms on the ideal As-terminated (111)B surface (Fig. 1(a)) are replaced by chalcogen atoms, as shown in Fig. 1(b)^12–15^. Similar to the case for the As-terminated Si(111) surface^16^, this atomic geometry has been believed to be highly passivated^17,18^. It has been demonstrated that the passivation of GaAs(111)B by S and Se makes it possible to heteroepitaxially grow layered transition-metal dichalcogenides, such as NbSe_2_ and MoSe_2_, despite a large lattice mismatch^19–21^. On the other hand, first-principles calculations have predicted that the surface-state density in the mid gap region is not reduced^14^. In addition, as we will show later, the model does not satisfy the so-called electron counting rule^22^, and, therefore, should be energetically unfavorable. Thus, the structure identification is not convincing, and more detailed and systematic studies are needed to establish whether the GaAs(111)B surface could be electronically passivated by chalcogen atoms.

Here we show that the Se atoms substitute the $\frac{3}{4}$ monolayer (ML) of As atoms at the outermost layer of GaAs(111)B (Fig. 1(f)). This atomic geometry eliminates all of unsaturated dangling bonds and surface-state bands in the mid-gap region, as confirmed by first-principles calculations. The proposed mixed Se/As-terminated model could account for all of experimental data from complementary experimental techniques of reflection high-energy electron diffraction (RHEED), scanning tunneling microscopy (STM), and x-ray photoelectron spectroscopy (XPS), and is found to be energetically stable. This is an unique example demonstrating an electronic passivation mechanism in a well-defined structure of S/Se-treated GaAs surface.

## Results and Discussion

**Figure 2:**
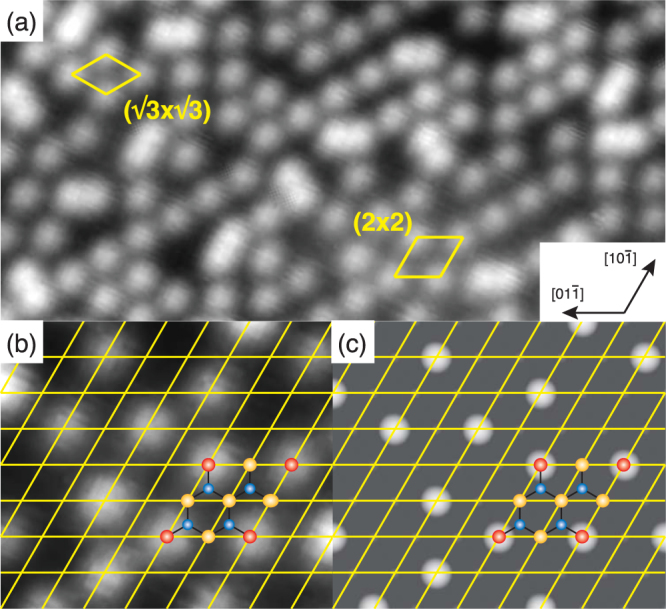
Typical filled-state STM image obtained from the Se-treated GaAs(111)B surface (**a**). Local (2 × 2) and ($\sqrt{3}\times \sqrt{3}$)-*R*30° units are indicated. (**b**) Magnified image of (**a**). The images were taken with a sample bias of −3.0 V. The image dimensions are 56 Å × 116 Å (**a**) and 27.7 Å × 32.0 Å (**b**). (**c**) Simulated images for the structure model shown in Fig. 1(f) using a filled-state bias of 3 V below the valence band maximum. The solid lines in (**b**) and (**c**) show the (1 × 1) lattice mesh. Ga, As, and Se atoms are indicated by blue, red, and yellow circles, respectively in (**b**) and (**c**).

Figure 2(a) shows a typical filled-state STM image of the Se-treated GaAs(111)B-(1 × 1) surface. Bright spots are randomly distributed, while (2 × 2) and ($\sqrt{3}\times \sqrt{3}$)-*R*30° units are locally observed as indicated in the figure. The essential features in STM images remained unchanged in the bias voltage ranges between −1.5 and −4.5 eV. The density of the bright spots is 0.2–0.25 ML per (1 × 1) unit. Thus, at first sight, the present STM observations are incompatible with the structure model with Se atoms replacing all of As atoms at the outermost surface layer (Fig. 1(b)).

To explain the observed STM features, we first examined the structure models with Se adatoms on T_4_ (Fig. 1(c)) and H_3_ (Fig. 1(d)) sites of the ideal As-terminated (111)B surfaces. On the ideal As-terminated surface, according to the electron counting model^22^, the dangling bond of the outermost As atom has $\frac{5}{4}$ electrons, which requires $\frac{3}{4}$ electrons to cause a lone pair state on the As atom. On the other hand, the adsorption of one Se atom on either the H_3_ or T_4_ site results in the excess $\frac{7}{4}$ electrons per single Se adatom, because $\frac{3}{4}\times 3$ electrons are consumed to form chemical bonds with three As atoms and two electrons are accommodated in the Se dangling bond $(6-2-\frac{3}{4}\times 3=\frac{7}{4})$. Thus, the adsorption of $\frac{3}{16}$ ML of Se could eliminate all of unsaturated dangling bonds by transferring the excess $(\frac{7}{4}\times \frac{3}{16})$ electrons at Se adatoms to the partially-filled dangling bonds ($\frac{3}{4}$ electrons) of remaining $\frac{7}{16}$ ML of surface As atoms $(\frac{3}{16}\times \frac{7}{4}-\frac{7}{16}\times \frac{3}{4}=0)$. Since the Se coverage of $\frac{3}{16}$ ML (=0.1875 ML) is rather close to the observed density of bright spots in STM image (0.2–0.25 ML), the adsorption on either site could account for the observed STM images on the assumption that Se adatoms manifest themselves as bright features in Fig. 1(a). However, these T_4_ and H_3_ models are inconsistent with the RHEED rocking-curve analysis, as we will show below.

**Figure 3:**
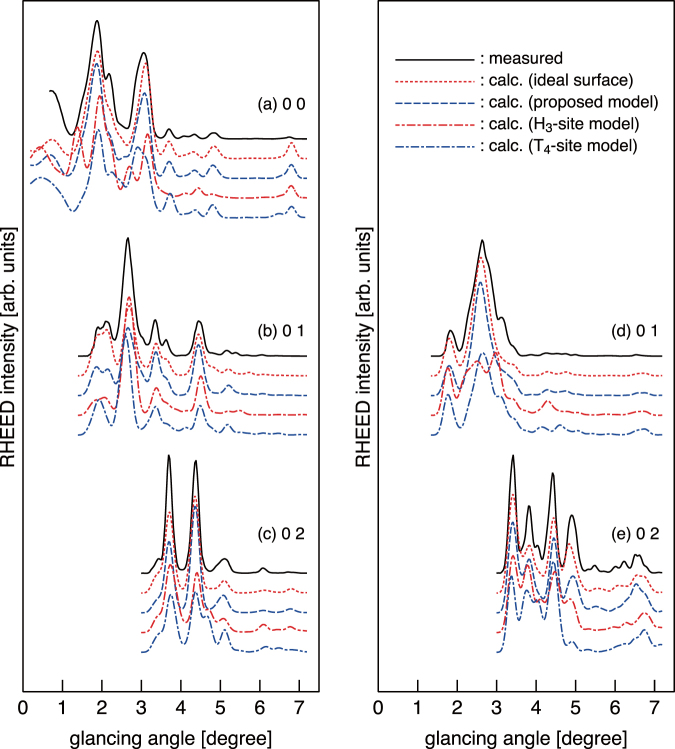
RHEED rocking curves (solid curves) measured from Se-terminated GaAs(111)B surface at room temperature. The dotted curves are calculated for the ideal As-terminated surface. The dashed curves are calculated using the atomic coordinates obtained by first-principles calculations for the structure model shown in Fig. 1(f). The dash-dotted curves are calculated for the optimized H_3_- and T_4_-site models.

Figure 3 shows RHEED rocking curves (solid curves) measured from the GaAs(111)B-(1 × 1)-Se surface, together with the calculated ones from the As-terminated ideal GaAs(111)B surface (dotted curves). In the present RHEED calculation, the atomic coordinates are fixed at their bulk positions. The agreement between the experiment and the calculation is excellent (*R*_min_ = 0.080), indicating that the Se-terminated (111)B surface has the atomic geometry quite similar to that of bulk-terminated (111)B surface. On the other hand, the T_4 _(Fig. 1(c)) and H_3_ (Fig. 1(d)) models show larger *R*-factors of 0.167 and 0.186, respectively, even after the structure optimization. As indicated by the dash-dotted curves in Fig. 3, neither of these models could reproduce the measured features. Thus, the T_4_ and H_3_-site models could be ruled out. We have also tested the on-top site model (Fig. 1(e)) proposed for the S-terminated GaAs(111)B^14^, and obtained a very large *R*-factor of 0.446.

Since the atomic scattering factors of Se and As are quite close, rocking curves calculated for the Se-terminated surface (Fig. 1(b)) are indistinguishable from those of the As-terminated surface (Fig. 1(a)). This motivated us to examine the possible coexistence of Se and As at the outermost layer. The As dangling bond of the As-terminated surface (Fig. 1(a)) lacks $\frac{3}{4}$ electrons per (1 × 1), while the Se dangling bond of the Se-terminated (1 × 1) surface (Fig. 1(b)) has excess $\frac{1}{4}$ electrons. Thus, the surface is electronically stabilized by replacing the $\frac{3}{4}$ of surface As atoms by Se atoms, leaving no unsaturated dangling bonds.

As mentioned earlier, the density of bright spots in the STM image (Fig. 2(a)) is 0.2–0.25 ML. Thus, if we assume that only As atoms are imaged as bright features in the observed STM image, the structure model terminated with 0.25 ML-As and 0.75 ML-Se atoms (Fig. 1(f)) could account for the observed STM image. To verify the hypothesis, we performed density functional theory (DFT) calculations and extracted simulated STM images using the Tersoff-Hamann formalism^23^. The calculations were carried out for the (2 × 2) units consisting of one As atom and three Se atoms at the outermost layer. Figure 2(b) and (c) compare the observed and simulated STM images. It is clearly seen that As atoms correspond to the bright spots and the Se atoms are observed as much less bright features. We simulated the STM images using bias voltages between −0.5 V and −3.0 V measured from the valence band maximum, and found that Se atoms are hardly imaged in the range of −0.5 to −2.0 V, and faintly visible at −2.5 V and −3V (See Supplementary Fig. S1). Thus, the combination of STM observations and simulations provides the strong evidence for the coexistence of Se and As atoms at the outermost layer.

The calculations were also performed for the larger unit cell of (4 × 4), in which 4 As atoms and 12 Se atoms are randomly arranged at the outermost layer. The simulated STM features and the atomic coordinates of the optimized structure are essentially the same as those for the (2 × 2) unit. Shown by dashed curves in Fig. 3 are RHEED rocking curves calculated using the atomic coordinates derived from the DFT calculations for the (4 × 4) unit (see Supplementary Information for coordinates). The *R* factors of the optimized (2 × 2) and (4 × 4) models are 0.075 and 0.080, respectively: the calculated curves reproduce well the most of features in measured curves.

RHEED calculations for the (2 × 2) and (4 × 4) unit cells were also carried out using 10 fractional-order and 11 integer-order reflections; (0 0), (0 $\pm \frac{1}{2}$), (0 ±1), …, (0 $\pm \frac{9}{2}$), and (0 ±5) for (2 × 2), and 30 fractional-order and 11 integer-order reflections; (0 0), (0 $\pm \frac{1}{4}$), (0 $\pm \frac{2}{4}$), …, (0 $\pm \frac{19}{4}$), and (0 ±5) for (4 × 4). We confirmed that the calculated intensities of integer-order reflections are almost all the same as those calculated without fractional-order reflections: *R* factors are in the narrow range of 0.075–0.083.

The proposed structure model (Fig. 1(f)) is stabilized by transferring $\frac{3}{4}$ electrons from three Se atoms to the As atom, so that both Se and As dangling bonds are fully occupied states. This charge transfer, however, seems to be inconsistent with the prediction based on the electronegativity: since the electronegativity values of Se and As are 2.4 and 2.0, respectively^24^, one may expect that more electrons are accumulated on the Se atom. In the optimized atomic geometry, As atoms are located at 0.28 Å higher than Se atoms. Accordingly, the bond angle of the surface As atom with its three Ga nearest neighbors is 118.05°, which is significantly larger than the value for the surface Se atom (110.74°). Nakamura *et al*. found that the orbital electronegativity for an *s*-*p* hybridized dangling bond increases as the angle to its back bonds is increased^25,26^. The resultant orbital electronegativity of the As dangling bond becomes larger than that of the Se atom in an almost ideal *sp*^3^ configuration, so that the charge transfer from Se to As is promoted.

**Figure 4:**
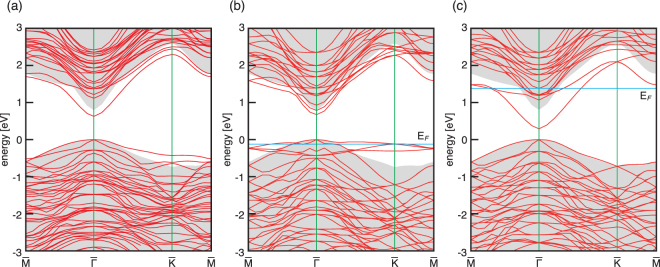
Energy-band structures of (**a**) mixed Se/As-terminated model, (**b**) As-terminated model, and (**c**) Se-terminated model for (2 × 2) unit cells. E_*F*_ shows a Fermi level. The energies refer to the valence band maximum of bulk GaAs. Gray regions indicate the projected bulk band structure. For the mixed Se/As-terminated model, the valence band below 0 eV is fully occupied, while the conduction one is completely unoccupied, showing the semiconducting nature. On the other hand, for the As-terminated and the Se-terminated models, the Fermi levels cross the valence and the conduction bands, respectively, showing the metallic nature of surfaces.

Figure 4(a–c) show the energy-band structures of the Se/As mixed model, As-terminated model, and Se-terminated model, respectively. The Se/As mixed model shows a semiconducting nature with a direct gap of 0.63 eV; the valence band below 0 eV is fully occupied, while the conduction one is completely unoccupied. On the other hand, the As- and Se-terminated surfaces are metallic: there are surface-state bands crossing the the Fermi level E_*F*_ located near the bulk valence-band maximum and conduction-band minimum on the As-terminated and Se-terminated surfaces, respectively. The surface-state bands for the As-terminated surface are originated from the As dangling bond, while those for the Se-terminated surface are associated with the Se-Ga antibonding states.

**Figure 5:**
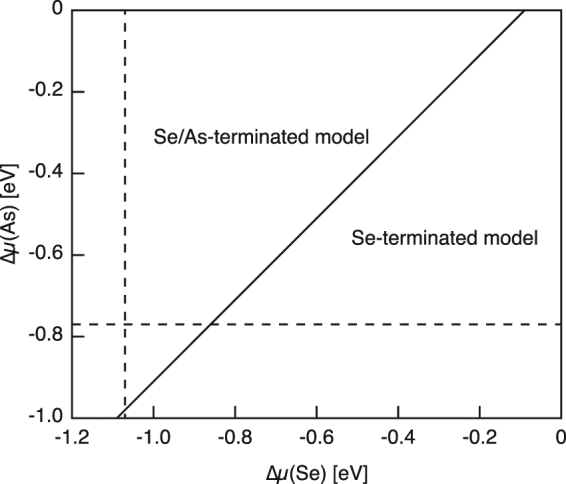
Phase diagram of the Se-treated (1 × 1) structures as functions of the relative potentials of As and Se with respect to their bulk phases. Vertical (horizontal) dashed line shows the chemical potential of Se (As) for the bulk Ga_2_Se_3_ (GaAs).

We have examined the relative stability of the proposed Se/As-terminated model. Because of the different numbers of Se and As atoms per unit cell, we have to take into account the chemical potentials of Se [Δ*μ*(Se)] and As [Δ*μ*(As)] to compare the total energies for different models. The phase diagram in dependence upon Δ*μ*(Se) and Δ*μ*(As) is shown in Fig. 5. While the As-terminated structure is unstable in the whole range of chemical potentials, the Se/As-terminated structure is the most stable for lower Δ*μ*(Se) and higher Δ*μ*(As), and the Se-terminated structure becomes energetically favorable as Δ*μ*(Se) is increased. This is in somewhat inconsistent with the experimental results: only the Se/As-terminated structure has been observed by STM. Since the vapor pressure of Se is extremely higher than that of As, it is plausible that the amount of excess Se on the surface is not enough to form the Se-terminated structure under the present experimental conditions.

We also calculated the formation energies of two types of (4 × 4) models consisting of randomly-arranged surface Se/As atoms and the model with regularly arranged Se/As atoms (see Fig. S2). While the regularly-arranged model is slightly more stable than randomly-arranged models, the difference in the energy is less than 6 meV/(1 × 1). This is in qualitative agreement with the coexistence of diverse atomic arrangements in observed STM images. In addition, we calculated RHEED intensities for several (4 × 4) atomic arrangements, and confirmed that the calculated RHEED intensities hardly depend on the distribution of surface Se and As atoms.

**Figure 6:**
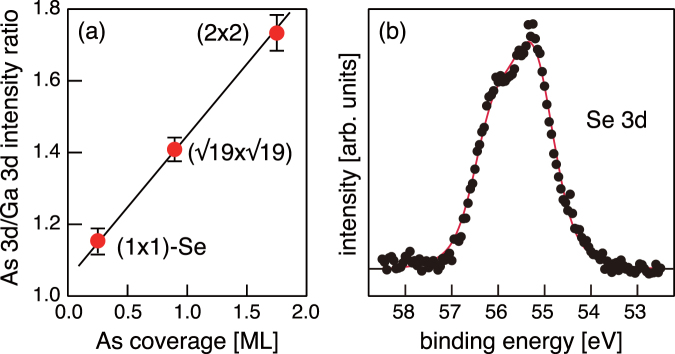
(**a**) Photoelectron intensity ratio of As 3*d*/Ga 3*d* for the As-rich (2 × 2), Ga-rich ($\sqrt{19}\times \sqrt{19}$), and Se-terminated (1 × 1) surfaces. (**b**) Se 3*d* photoelectron spectrum for the Se-treated (1 × 1) surface. The result of the fitting is shown by the solid curve.

Figure 6(a) shows photoelectron intensity ratios of As 3*d*/Ga 3*d* for the clean surfaces of As-rich (2 × 2) and Ga-rich $(\sqrt{19}\times \sqrt{19})$-*R*23.4° and Se-treated (1 × 1) surfaces of GaAs(111)B. The data are plotted as a function of the As coverage of the structure models. The As coverage of the proposed Se/As-terminated model (Fig. 1(f)) is 0.25 ML, while the values of the $(\sqrt{19}\times \sqrt{19})$ and (2 × 2) reconstructions are 0.894 ML^27^ and 1.75 ML^28^, respectively. The intensity ratio linearly increases with surface As coverage, further supporting the validity of the proposed model. Another important implication is that Se atoms do not substitute the As atoms at subsurface layers. Although previous studies have shown that Se and S atoms replace As atoms in the subsurface layers of GaAs(111)B^13,15,29^, such an atomic replacement produces excess charge in the surface layers, making the surface unstable. Considering that the Se 3*d* photoelectron spectra for the Se-terminated (111)B surface could be well fitted with a single component, as shown in Fig. 6(b), we conclude that the Se-As exchange reaction at subsurface layers is negligible.

Finally, we will briefly discuss the effect of the initial surface reconstruction on the Se termination. We found that the Se treatment on the $(\sqrt{19}\times \sqrt{19})$ surface results in multilayered morphology as compared to the atomically flat surface formed on the initial (2 × 2) surface^30^. The (2 × 2) structure has As trimers on the complete Ga-As bilayer, while $\sqrt{19}$ surface has a hexagonal ring structure consisting of As atoms on the incomplete Ga layer ($\frac{12}{19}$ ML). Thus the removal of $\frac{12}{19}$ ML-Ga or the incorporation of $\frac{7}{19}$ ML-Ga is required for the formation of the flat Se-terminated (1 × 1) structure on the initial $\sqrt{19}$ surface. On the other hand, the atomically flat Se-terminated (1 × 1) structure is easily formed on the (2 × 2) surface by simply substituting As atoms by Se atoms together with the desorption of excess As atoms.

## Conclusions

We have revisited a long-standing question on the atomic and electronic structures of the Se-treated GaAs(111)B-(1 × 1) surface. The combination of STM observations and simulations enables us to discriminate two different atomic species of Se and As at the outermost layer. The structure model has been proposed, in which 0.75 ML of surface As atoms are substituted by the Se atoms and the remaining As atoms (0.25 ML) are located at the vertical position 0.28 Å higher than that of the substitutional Se atoms. This atomic geometry promotes the charge transfer from Se to As, eliminating all of unsaturated dangling bonds. The resultant surface is electronically passivated with no surface states in the mid-gap region.

## Methods

### Experiments

The experiments were performed using a system of interconnecting ultra-high vacuum (UHV) chambers for the III-V molecular-beam epitaxy (MBE) growth, for the Se treatments, and for online characterization by means of STM and XPS^30,31^. The III-V MBE chamber is equipped with RHEED apparatus for rocking-curve measurements. The clean GaAs(111)B-(2 × 2) surfaces were obtained by growing an undoped layer (0.5 *μ*m) on a thermally cleaned Si-doped GaAs(111)B substrate^32^. The clean GaAs samples were transferred to another UHV chamber for the Se treatments via UHV transfer modules (<2 × 10^−10^ Torr), and were exposed to the Se molecular beam at 300 °C, and then annealed at 530 °C under the Se flux to form the atomically flat Se-terminated (1 × 1) surface^30^. The beam-equivalent pressure of Se was 7 ~9 × 10^−8^ Torr.

All the STM images were collected at RT in the constant current mode with a tunneling current of 0.1 nA and a sample voltage of −1.5 ~−4.5 V. XPS measurements were performed using monochromatic Al K *α* radiation (1486.6 eV). Photoelectrons were detected at an angle of 35° from the surface. The Se 3*d*, As 3*d*, and Ga 3*d* spectra were measured and fitted using a Voigt function with the ratio of Gaussian to Lorentzian components fixed at 2.5. The Lorentzian (Gaussian) widths of Ga 3*d*, As 3*d*, and Se 3*d* obtained from the fit are 0.29 (0.59) eV, 0.31 (0.64) eV, and 0.40 (0.83) eV, respectively. These values are in good agreement with those reported earlier^33,34^. Peak separations of 0.85 eV, 0.68 eV, and 0.54 eV are assumed for the 5/2 and 3/2 spin-orbit components of Se 3*d*, As 3*d*, and Ga 3*d*, respectively.

RHEED rocking curves of (0 0), (0 ±1), and (0 ±2) spots were measured along the [10 $\overline{1}$] direction. The energy of the incident electrons was set at 15 keV. The glancing angle of the incident electron beam was typically changed from 0.5 ° to 7.2 ° with intervals of 0.025 °. RHEED intensities were calculated by the multislice method^35,36^ using 11 integer-order reflections. Parameters used in the calculations, such as elastic and inelastic scattering potentials, and thermal vibrations, were derived as described elsewhere^37^. To quantify the agreement between the experiments and calculations, the *R* factor defined in ref.^38^ was used.

### Calculations

We performed first-principles calculations^39,40^ within the DFT^41^ in the generalized gradient approximation^42^. The potentials are described by ultrasoft pseudopotentials in the Vanderbilt form^39^. The valence electron configurations are 4*s*^2^4*p*^1^ for Ga, 4*s*^2^4*p*^3^ for As, and 4*s*^2^4*p*^4^ for Se. The calculated lattice constant of GaAs is 5.734 Å, which is close to the experimental value of 5.6538 Å. A slab geometry was used for the simple calculation, which has the supercell consisting of 10 atomic layers and of vacuum region (20 Å in thickness). The back side of the slab is terminated with fictitious H atoms, which eliminate artificial dangling bonds and prevent it from coupling with the front side. The wave functions were expanded by the plane wave basis set with a cutoff energy of 36 Ry. 4 × 4 × 1 *k* points were used for the integration in *k* space in the Brillouin zone for the (2 × 2) unit cell.

### Data availability

The data that support the findings of this study are available from the corresponding author upon reasonable request.

## Electronic supplementary material

dataset2

dataset1

Supplementary Information
