# Supplementary material for: Atomic structure and passivated nature of the Se-treated GaAs(111)B surface
Source: Sci Rep. 2018 Jan 19;8:1220. doi: 10.1038/s41598-018-19560-2 (PMC5775421; doi:10.1038/s41598-018-19560-2)
Supplement: Supplementary file 3 — Supplementary Information [file 41598_2018_19560_MOESM3_ESM.doc]

Supplementary Information

Atomic structure and passivated nature of the Se-treated GaAs(111)B surface

Akihiro Ohtake,^*,†^ Shunji Goto, ^‡^ and Jun Nakamura^‡^

*^†^National Institute for Materials Science (NIMS), Tsukuba 305-0044, Japan*

*^‡^Department of Engineering Science, The University of Electro-Communications (UEC-Tokyo), Chofu, Tokyo 182-8585, Japan*

**Corresponding Author:** [OHTAKE.Akihiro@nims.go.jp](mailto:OHTAKE.Akihiro@nims.go.jp)

**

**

**Figure S1.** Simulated STM images for the (2x2) unit cell of the mixed Se/As-terminated GaAs(111)B model. The bias voltages are given with respect to the valence band maximum.


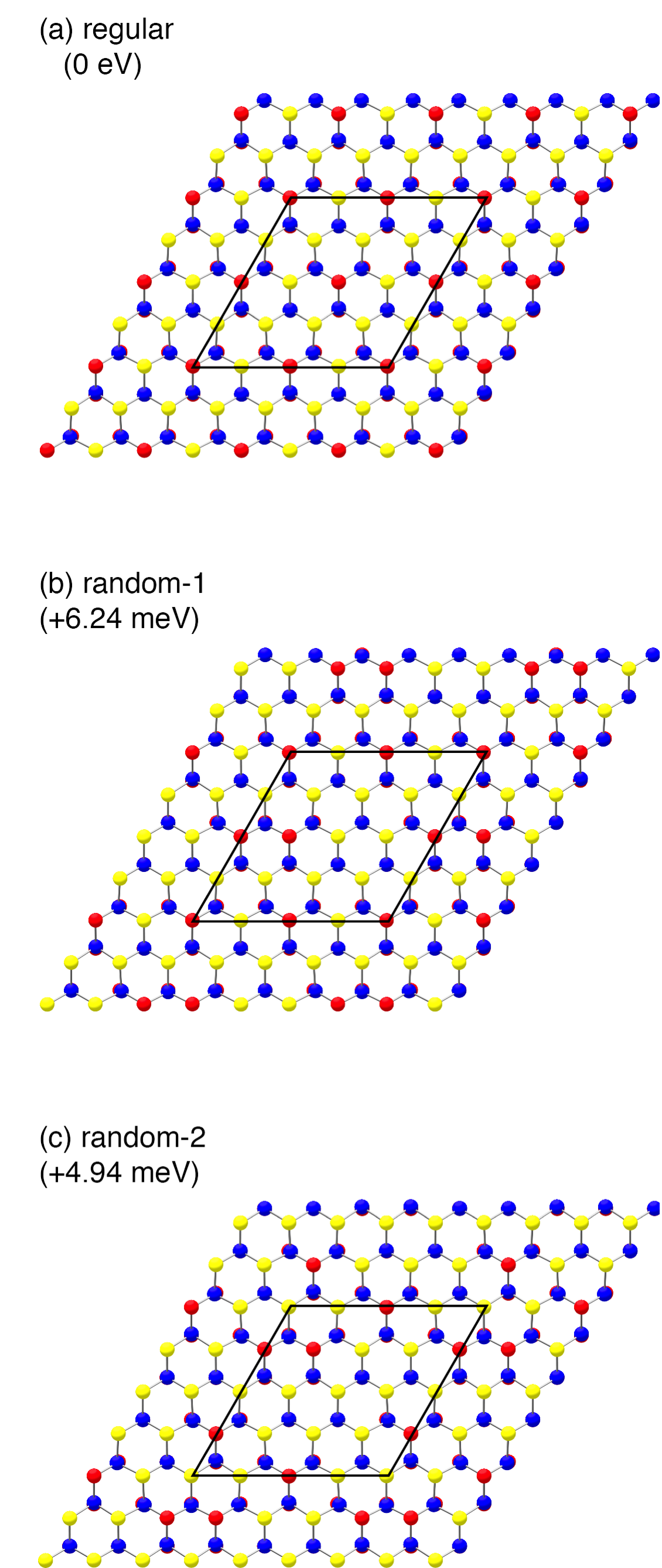


**Figure S2.** Structure models of Se/As terminated surface having different atomic configurations in the (4x4) unit cell. Surface Se/As atoms are regularly arranged in the model (a). The models (b) and (c) consist of randomly-arranged surface As/Se atoms. The (4x4) unit cell is indicated in each model.


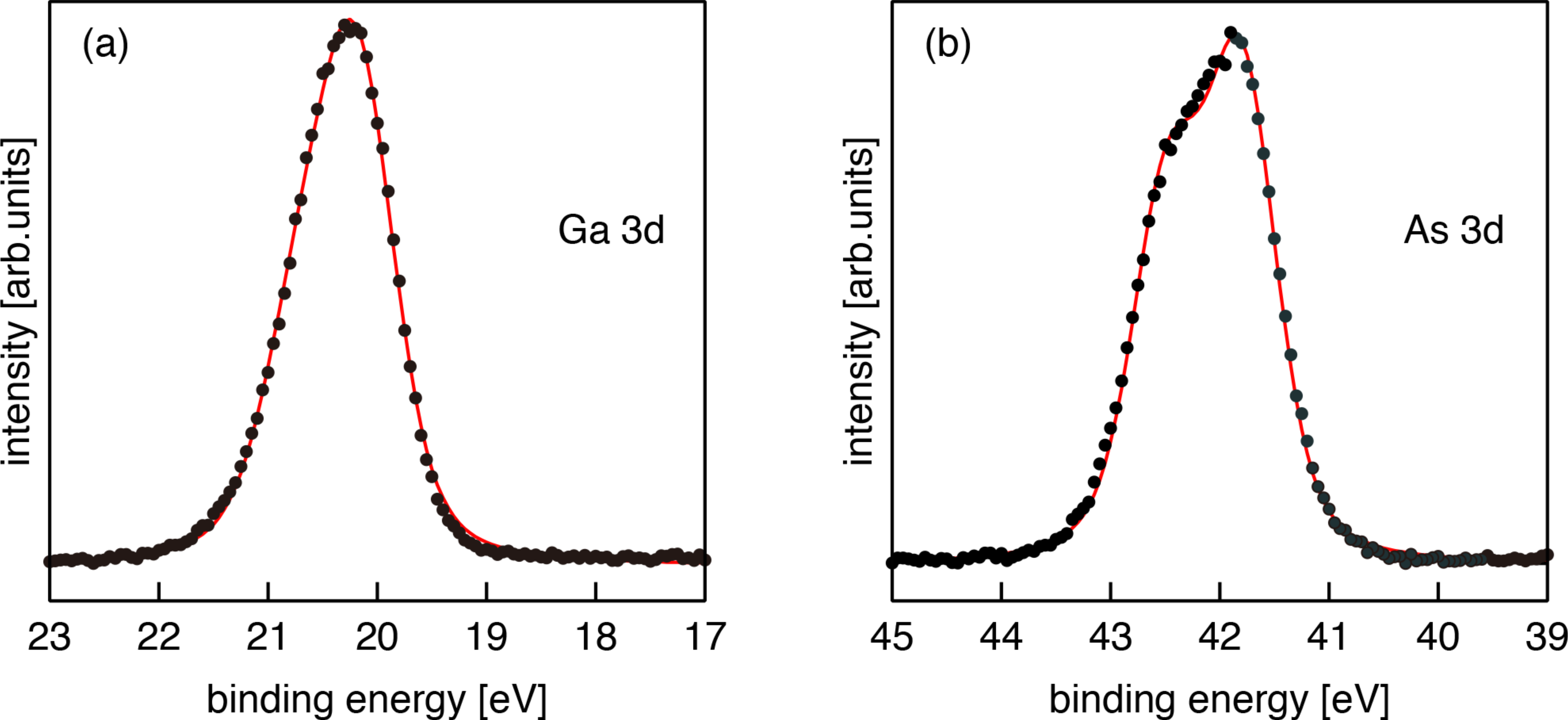


**Figure S3.** (a) Ga 3d and (b) As 3d photoelectron spectra for the Se-treated (1x1) surface. The results of the fitting are shown by the solid curves.
